# Supplementary material for: Content-rich biological network constructed by mining PubMed abstracts
Source: BMC Bioinformatics. 2004 Oct 8;5:147. doi: 10.1186/1471-2105-5-147 (PMC528731; doi:10.1186/1471-2105-5-147)
Supplement: Additional File 2 — The original results of the above study (non-essential files are deleted to keep the file size under the limit set by BMC bioinformatics). [file 1471-2105-5-147-S2.bz2 › chilibotAdditionalFile2/dip50/657ID10353244E11048/html/KPNB1_KPNA2.html]

 


 **KPNB1** and **KPNA2** 
  
Found 172 abstracts in PubMed, retrieved 50.  
 

 What does Google say? 
 PDF only 
| .edu only 

---

**Interactive relationship** (e.g. stimulation, inhibition, etc)

**Neutral relationship**- The Saccharomyces cerevisiae NPC is comprised of 30 nucleoporins Nups, 13 of which contain phenylalanine glycine repeats FG Nups that bind karyopherins and facilitate the transport of karyopherin  [ **KPNA2** ]   [ **KPNB1** ]  cargo complexes.  Ref: 12604785 Proc Natl Acad Sci U S A, 2003
- While performing a yeast two hybrid library screen to uncover novel PP2A interacting proteins, we discovered a specific interaction between a member of the importin beta karyopherin  [ **KPNA2** ]   [ **KPNB1** ]  beta superfamily, importin 9, and the A subunit of PP2A PR65.  Ref: 12670497 Biochem Biophys Res Commun, 2003
- Genetic and physical interactions between Srp1p and VDE imply direct involvement of karyopherin  [ **KPNA2** ]   [ **KPNB1** ]  mediated nuclear transport in this process.  Ref: 12588991 Mol Cell Biol, 2003
- We also show that both L and D amino acid containing peptides bind well to karyopherin  [ **KPNA2** ]   [ **KPNB1** ]  alpha 2.  Ref: 12504098 Biochem Biophys Res Commun, 2003
- ... the L amino acid peptides bind more efficiently than the D amino acid peptide to karyopherin  [ **KPNA2** ]   [ **KPNB1** ]  alpha1.  Ref: 12504098 Biochem Biophys Res Commun, 2003
- We demonstrate that Nap1p can bind directly to Kap114p, the primary karyopherin  [ **KPNA2** ]   [ **KPNB1** ]  importin responsible for the nuclear import of H2A and H2B.  Ref: 12456659 EMBO J, 2002
- An intracellular targeted NLS peptide inhibitor of karyopherin  [ **KPNA2** ]   [ **KPNB1** ]  alpha NF kappa B interactions.  Ref: 12504098 Biochem Biophys Res Commun, 2003
- Karyopherin  [ **KPNA2** ]   [ **KPNB1** ]  alpha2 escorts proteins into the nucleus via interaction with a nuclear localization sequence NLS composed of short stretches of basic amino acids.  Ref: 11882654 J Biol Chem, 2002
- These karyopherin  [ **KPNA2** ]   [ **KPNB1** ]  structures provide valuable insights into understanding the molecular mechanism of nuclear import, especially substrate recognition, substrate release by GTPase and interactions with the nuclear pore complex.  Ref: 11751052 Curr Opin Struct Biol, 2001
- Transport of macromolecules across the nuclear pore complex NPC occurs in seconds and involves assembly of a karyopherin  [ **KPNA2** ]   [ **KPNB1** ] .cargo complex and docking to the NPC, translocation of the complex across the NPC via interaction with nucleoporins Nups, and dissociation of the complex in the nucleoplasm.  Ref: 11867631 J Biol Chem, 2002
- The predicted NLS1 KRKR was essential for physical interaction with karyopherin  [ **KPNA2** ]   [ **KPNB1** ]  alpha2 in glutathione S transferase pull down assay, and its mutation resulted in decreased nuclear import of PLAG1.  Ref: 11882654 J Biol Chem, 2002
- Physical interaction between PLAG1 and karyopherin  [ **KPNA2** ]   [ **KPNB1** ]  alpha2 was confirmed by an in vitro glutathione S transferase pull down assay.  Ref: 11882654 J Biol Chem, 2002
- A subgroup of the karyopherin  [ **KPNA2** ]   [ **KPNB1** ]  beta also called importin beta protein that includes budding yeast Kap104 and human transportin karyopherin  [ **KPNA2** ]   [ **KPNB1** ]  beta2 is reported to function as a receptor for the transport of mRNA binding proteins into the nucleus.  Ref: 12524331 Genetics, 2002
- Nuclear localization of the tyrosine kinase Itk and interaction of its SH3 domain with karyopherin  [ **KPNA2** ]   [ **KPNB1** ]  alpha Rch1alpha.  Ref: 11581171 Int Immunol, 2001
- The results demonstrate a global role for Gsp1p GTP on karyopherin  [ **KPNA2** ]   [ **KPNB1** ]  nucleoporin interactions and provide a rudimentary map of the routes that karyopherins take as they cross the nuclear pore complex.  Ref: 11387327 J Biol Chem, 2001
- **KPNA2** , as part of a karyopherin  [ **KPNA2** ]   [ **KPNB1** ]  alpha beta heterodimer, directly binds to the NLS of proteins and functions as an adaptor that binds NLS containing proteins via karyopherin  [ **KPNA2** ]   [ **KPNB1** ]  beta to the nuclear pore complex.  Ref: 11735022 Hum Genet, 2001

**Non-interactive relationship** (e.g. studied together, co-existance, homology, etc.)

- Prospective mimetics with linkers of, 10, or 12 atoms were synthesized and compared in a competition assay for binding to the nuclear import receptor subunit karyopherin  [ **KPNA2** ]   [ **KPNB1** ]  alpha, showing the 10 atom linker to be best and shorter ones ineffective.  Ref: 12841749 Org Lett, 2003
- We report here that the karyopherin  [ **KPNA2** ]   [ **KPNB1** ]  Mtr10p is required for the normal accumulation of mature Tlc1 and its proper localization to the nucleus.  Ref: 12167699 Mol Cell Biol, 2002
- We found that the karyopherin  [ **KPNA2** ]   [ **KPNB1** ]  beta related export receptor CRM1 competes with the Tap transport domain for binding to Nup214 but not for binding to Nup62 or Nup153, suggesting that the Tap and CRM1 nuclear export pathways converge at the cytoplasmic periphery of the nuclear pore complex.  Ref: 11551912 J Biol Chem, 2001
- Furthermore, we demonstrate that Nap1p promotes the association of the H2A and H2B NLSs specifically with the karyopherin  [ **KPNA2** ]   [ **KPNB1** ]  Kap114p.  Ref: 12456659 EMBO J, 2002
- The amount of inactive karyopherin  [ **KPNA2** ]   [ **KPNB1** ]  alpha2 receptor reduced, in a dose dependent manner, the GLUT2 and liver pyruvate kinase mRNA levels by competition with endogenous active receptor.  Ref: 11988093 Biochem J, 2002
- We show that the yeast karyopherin  [ **KPNA2** ]   [ **KPNB1** ]  Kap121p functions in the targeting and assembly of the nucleoporin Nup53p into NPCs by recognizing a nuclear localization signal NLS in Nup53p.  Ref: 12403813 J Cell Biol, 2002
- Consistent with the hypothesis that Crm1 is not the only karyopherin  [ **KPNA2** ]   [ **KPNB1** ]  cofactor for MS2 Ran mediated mRNA export, we show that not only Crm1 but also CAS, transportin, importin beta and exportin t can all export mRNA from the nucleus when tethered via the MS2 RNA binding domain.  Ref: 11911364 RNA, 2002
- The present study suggests that, in concert with glucose metabolism, karyopherin  [ **KPNA2** ]   [ **KPNB1** ]  alpha2 transmits a signal to the nucleus to regulate glucose sensitive gene expression.  Ref: 11988093 Biochem J, 2002
- Results from this study showed that fertilized porcine embryos injected with double stranded RNA dsRNA corresponding to the importin alpha homologue karyopherin  [ **KPNA2** ]   [ **KPNB1** ]  alpha3 had significantly fewer nuclei following four days of culture than did embryos injected with dsRNA for another importin alpha homologue, karyopherin  [ **KPNA2** ]   [ **KPNB1** ]  alpha2, or two control groups.  Ref: 12548662 Mol Reprod Dev, 2003
- Crm1 is a member of the karyopherin  [ **KPNA2** ]   [ **KPNB1** ]  family of nucleocytoplasmic transport receptors and mediates the export of proteins from the nucleus by forming a ternary complex with cargo and Ran GTP.  Ref: 11425870 J Cell Biol, 2001
- Karyopherin  [ **KPNA2** ]   [ **KPNB1** ]  mediated nuclear import of the homing endonuclease VMA1 derived endonuclease is required for self propagation of the coding region.  Ref: 12588991 Mol Cell Biol, 2003
- Recently, crystal structures of karyopherin  [ **KPNA2** ]   [ **KPNB1** ]  alpha and its complexes with nuclear localization signal peptides, a karyopherin  [ **KPNA2** ]   [ **KPNB1** ]  beta2 Ran complex and complexes of full length and fragments of karyopherin  [ **KPNA2** ]  beta1  [ **KPNB1** ]  with import substrates, Ran and nucleoporins have been solved.  Ref: 11751052 Curr Opin Struct Biol, 2001
- Analysis of its sequence specific interacting protein indicated that JKTBP nuclear import is mediated by the receptor transportin 1 karyopherin  [ **KPNA2** ]   [ **KPNB1** ]  beta2.  Ref: 11705999 J Biol Chem, 2002
- RNA export mediated by the minihelix motif is Ran dependent, which indicates the involvement of a karyopherin  [ **KPNA2** ]   [ **KPNB1** ]  related factor exportin that remained to be determined.  Ref: 12509441 J Biol Chem, 2003
- In contrast, the overexpression of karyopherin  [ **KPNA2** ]   [ **KPNB1** ]  alpha2 did not significantly stimulate GLUT2 and liver pyruvate kinase mRNA accumulation in green fluorescent protein sorted cells.  Ref: 11988093 Biochem J, 2002
- The peptide receptor complex associates with karyopherin  [ **KPNA2** ]   [ **KPNB1** ] , translocates through the nuclear pore, and can be observed in the inner nuclear matrix and at the periphery of heterochromatin of the nucleus.  Ref: 11890982 Brain Res Brain Res Rev, 2002
- We found that HPV16 E6 interacted with the karyopherin  [ **KPNA2** ]   [ **KPNB1** ]  Kap alpha2 adapter and could enter the nucleus via a classical Kap alpha2beta1 mediated pathway.  Ref: 12551970 J Virol, 2003
- We report the development of a simple and highly efficient lipofection method for primary embryonic rat hippocampal neurons up to 25% transfection that exploits the M9 sequence of the non classical nuclear localization signal of heterogeneous nuclear ribonucleoprotein A1 for targeting beta 2 karyopherin  [ **KPNA2** ]   [ **KPNB1** ]  transportin 1.  Ref: 12044466 Neuroscience, 2002
- Developmental regulation of the heat shock response by nuclear transport factor karyopherin  [ **KPNA2** ]   [ **KPNB1** ]  alpha3.  Ref: 11546751 Development, 2001
- Protein cargoes that contain a classical nuclear localization signal NLS are transported into the nucleus through binding to a heterodimeric receptor comprised of importin karyopherin  [ **KPNA2** ]   [ **KPNB1** ]  alpha and beta.  Ref: 12672802 J Biol Chem< MedlineTA>J Biol Chem, 2001
- We describe the identification and characterization of a novel nuclear localization sequence NLS in Crz1p, which requires both basic and hydrophobic residues for activity, and show that the karyopherin  [ **KPNA2** ]   [ **KPNB1** ]  Nmd5p is required for Crz1p nuclear import.  Ref: 11535618 J Cell Biol, 2001
- We report a physical and functional association between the Tec family tyrosine kinase Itk Emt Tsk and the nuclear import chaperone karyopherin  [ **KPNA2** ]   [ **KPNB1** ]  alpha Rch1alpha in human T cells.  Ref: 11581171 Int Immunol, 2001
- Exportin 5, a novel karyopherin  [ **KPNA2** ]   [ **KPNB1** ] , mediates nuclear export of double stranded RNA binding proteins.  Ref: 11777942 J Cell Biol, 2002
- Nuclear import of many cellular and viral proteins is mediated by short nuclear localization signals NLS that are recognized by intracellular receptor proteins belonging to the importin karyopherin  [ **KPNA2** ]   [ **KPNB1** ]  alpha and beta families.  Ref: 11796712 J Biol Chem, 2002
- Identification of a karyopherin  [ **KPNA2** ]   [ **KPNB1** ]  alpha 2 recognition site in PLAG1, which functions as a nuclear localization signal.  Ref: 11882654 J Biol Chem, 2002
- The Ran protein regulates nucleocytoplasmic transport mediated by the karyopherin  [ **KPNA2** ]   [ **KPNB1** ]  family of nuclear transport factors.  Ref: 11911364 RNA, 2002
- This karyopherin  [ **KPNA2** ]   [ **KPNB1** ]  mediated function can also be performed by the Kap95p Kap60p complex if the Kap121p binding domain of Nup53p is replaced by a classical NLS, suggesting a more general role for karyopherins in NPC assembly.  Ref: 12403813 J Cell Biol, 2002
- Second, the import of neoglycoproteins was not affected by microinjection of anti nuclear import factor importin karyopherin  [ **KPNA2** ]   [ **KPNB1** ]  beta antibodies while the NLS dependent transport was completely abolished.  Ref: 12672698 Glycobiology< MedlineTA>Glycobiology, 2002
- By using the srp1 49 mutant which is deficient in nuclear import of cargos with classical nuclear localization sequences cNLS, we show that proteasome precursor complexes associate with importin karyopherin  [ **KPNA2** ]   [ **KPNB1** ]  alphabeta, the cNLS receptor,.  Ref: 11922673 J Mol Biol, 2002
- In contrast, predicted NLS2 of PLAG1 KPRK was not involved in karyopherin  [ **KPNA2** ]   [ **KPNB1** ]  alpha2 binding nor in its nuclear import.  Ref: 11882654 J Biol Chem, 2002
- Proteins that contain a classical nuclear localization signal NLS are recognized in the cytoplasm by a heterodimeric import receptor composed of importin karyopherin  [ **KPNA2** ]   [ **KPNB1** ]  alpha and beta.  Ref: 12486120 J Biol Chem, 2003
- Using the 1.6 kb cDNA fragment as bait, the following interacting proteins were identified Ran, transportin karyopherin  [ **KPNA2** ]   [ **KPNB1** ]  beta2, two proteins related to the nucleocytoplasmic transporter and aF10 protein.  Ref: 11774384 Mol Reprod Dev, 2002
- Treatment with the karyopherin  [ **KPNA2** ]   [ **KPNB1** ]  specific inhibitor leptomycin B was toxic to T. brucei and eliminated the cytoplasmic SL RNA, suggesting that cytoplasmic SL RNA was dependent on the nuclear exporter exportin 1 XPO1.  Ref: 12684371 Eukaryot Cell, 2003
- Using that domain as bait in a yeast two hybrid system we now report the identification and cloning of a Drosophila nuclear transport protein karyopherin  [ **KPNA2** ]   [ **KPNB1** ]  alpha3 dKap alpha3.  Ref: 11546751 Development, 2001
- Genomic structure of karyopherin  [ **KPNA2** ]   [ **KPNB1** ]  alpha2  **KPNA2**  within a low copy repeat on chromosome 17q23 q24 and mutation analysis in patients with Russell Silver syndrome.  Ref: 11735022 Hum Genet, 2001
- In the present work, we have characterized the function of the karyopherin  [ **KPNA2** ]   [ **KPNB1** ]  Msn5p in the control of the cell cycle of Saccharomyces cerevisiae.  Ref: 12697814 Mol Cell Biol, 2003
- TCR CD3 stimulation of Jurkat T cells resulted in increased Itk Rch1alpha complex formation, recruitment of karyopherin  [ **KPNA2** ]   [ **KPNB1** ]  beta to the protein complex and Rch1alpha tyrosine phosphorylation.  Ref: 11581171 Int Immunol, 2001
- Most of these receptors belong to one large family of proteins, all of which share homology with the protein import receptor importin beta also named karyopherin  [ **KPNA2** ]   [ **KPNB1** ]  beta.  Ref: 11423015 Genome Biol, 2001
- Karyopherin  [ **KPNA2** ]   [ **KPNB1** ]  alpha2 a control step of glucose sensitive gene expression in hepatic cells.  Ref: 11988093 Biochem J, 2002
- Most nucleocytoplasmic traffic through the nuclear pore complex is mediated by soluble receptors of the importin exportin or karyopherin  [ **KPNA2** ]   [ **KPNB1** ]  family.  Ref: 12637516 J Biol Chem< MedlineTA>J Biol Chem, 2002
- In the present study, by interaction with the GLUT2 loop, we have cloned the rat karyopherin  [ **KPNA2** ]   [ **KPNB1** ]  alpha2, a receptor involved in nuclear import.  Ref: 11988093 Biochem J, 2002
- A role for karyopherin  [ **KPNA2** ]   [ **KPNB1** ]  alpha2 in the transcription of two glucose sensitive genes was investigated by transfection of native and inactive green fluorescent protein karyopherin  [ **KPNA2** ]   [ **KPNB1** ]  alpha2 in GLUT2 expressing hepatoma cells.  Ref: 11988093 Biochem J, 2002
- The transitory tethering of karyopherin  [ **KPNA2** ]   [ **KPNB1** ]  alpha2 to GLUT2 at the plasma membrane might indicate that the receptor can load the cargo to be imported locally.  Ref: 11988093 Biochem J, 2002
- Here, we show that these necleoporins can be isolated from yeast extracts by affinity chromatography on karyopherin  [ **KPNA2** ]   [ **KPNB1** ]  Kap95p coated beads.  Ref: 11535617 J Cell Biol, 2001
- The NLS of SXR is characterized as the bipartite type, and is recognized by the three molecular species of importin alpha Rch1  [ **KPNA2** ]  PTAC58  [ **KPNA2** ] , NPI1, and Qip1, in the presence of PTAC97  [ **KPNB1** ]  of importin beta to target the nuclear pore.  Ref: 12606758 Mol Pharmacol, 2003
- Furthermore, the transport defective 31 allele of the karyopherin  [ **KPNA2** ]   [ **KPNB1** ]  SRP1, but not the transport competent 49 allele, exhibited a tab phenotype.  Ref: 11914130 BMC Genet, 2002
- In either case, the Ran.GTP is then transported to the cytoplasm by the karyopherin  [ **KPNA2** ]   [ **KPNB1** ] , where it is hydrolyzed to Ran.GDP.  Ref: 11911364 RNA, 2002
- A library of 30 mutants of the bipartite NLS from Xenopus laevis nucleoplasmin containing a novel, photoisomerizable amino acid was prepared by parallel, solid phase synthesis and screened in vitro for binding to the nuclear import receptor karyopherin  [ **KPNA2** ]   [ **KPNB1** ]  alpha, which mediates the nuclear import of cellular proteins.  Ref: 11711297 Bioorg Med Chem, 2001
- In nonpreferred nitrogen or nitrogen starvation, Gln3p is dephosphorylated and imported into the nucleus via karyopherin  [ **KPNA2** ]   [ **KPNB1** ]  alpha Srp1p.  Ref: 12624103 J Biol Chem, 2003
- Thirteen nucleoporins that contain FG peptide repeats FG Nups are proposed to function as stepping stones in karyopherin  [ **KPNA2** ]   [ **KPNB1** ]  mediated transport pathways.  Ref: 11387327 J Biol Chem, 2001
- Reconstitution assays revealed that only precursor complexes are targeted to the nuclear envelope NE by karyopherin  [ **KPNA2** ]   [ **KPNB1** ]  alphabeta.  Ref: 11922673 J Mol Biol, 2002
- Using the yeast two hybrid system, we identified karyopherin  [ **KPNA2** ]   [ **KPNB1** ]  alpha2 as a PLAG1 interacting protein.  Ref: 11882654 J Biol Chem, 2002
- A photoregulated ligand for the nuclear import receptor karyopherin  [ **KPNA2** ]   [ **KPNB1** ]  alpha.  Ref: 11711297 Bioorg Med Chem, 2001
- These observations indicate that the nuclear import of PLAG1 is governed by its zinc finger domain and by NLS1, a karyopherin  [ **KPNA2** ]   [ **KPNB1** ]  alpha2 recognition site.  Ref: 11882654 J Biol Chem, 2002
- Accelerating the rate of disassembly of karyopherin  [ **KPNA2** ]   [ **KPNB1** ] .cargo complexes.  Ref: 11867631 J Biol Chem, 2002
- Proteins of the karyopherin  [ **KPNA2** ]   [ **KPNB1** ]  alpha and karyopherin  [ **KPNA2** ]   [ **KPNB1** ]  beta families play a central role in nucleocytoplasmic transport.  Ref: 11751052 Curr Opin Struct Biol, 2001
- Kap123p is a yeast beta karyopherin  [ **KPNA2** ]   [ **KPNB1** ]  that imports ribosomal proteins into the nucleus prior to their assembly into preribosomal particles.  Ref: 12612077 Mol Cell Biol, 2003
- each bound 6 10 different karyopherin  [ **KPNA2** ]   [ **KPNB1** ]  betas, including importins as well as exportins.  Ref: 11387327 J Biol Chem, 2001
- Karyopherin  [ **KPNA2** ]  beta1  [ **KPNB1** ]  levels do not change with age and present very little variation among donors.  Ref: 12051719 Biochem Biophys Res Commun, 2002
- Our results indicate that VIP1 is imported into the plant cell nucleus via the karyopherin  [ **KPNA2** ]   [ **KPNB1** ]  alphadependent pathway.  Ref: 12124400 Proc Natl Acad Sci U S A, 2002
- In that way, Nup1p, Nup2p, Cse1p, and Gsp1p may function as karyopherin  [ **KPNA2** ]   [ **KPNB1** ]  release factors or KaRFs in the nuclear basket structure of the S. cerevisiae NPC.  Ref: 11867631 J Biol Chem, 2002
- The specificity of the binding was restricted to GLUT2, and not GLUT1 or GLUT4, and to karyopherin  [ **KPNA2** ]   [ **KPNB1** ]  alpha2, not alpha1.  Ref: 11988093 Biochem J, 2002
- Age dependent decreases in the protein concentrations of the nucleocytoplasmic transport factors karyopherin  [ **KPNA2** ]   [ **KPNB1** ]  alpha2, CAS, and RanBP1 were found by comparing fibroblast cultures obtained from young, mature, and old human donors.  Ref: 12051719 Biochem Biophys Res Commun, 2002
- Human karyopherin  [ **KPNA2** ]   [ **KPNB1** ]  alpha2  **KPNA2** , a member of the karyopherin  [ **KPNA2** ]   [ **KPNB1** ]  alpha family, plays a key role in the nuclear import of proteins with a classical nuclear localization signal NLS.  Ref: 11735022 Hum Genet, 2001
- The dynamics of karyopherin  [ **KPNA2** ]   [ **KPNB1** ]  mediated nuclear transport.  Ref: 11716302 Biochem Cell Biol, 2001
- All non coding RNAs are now known to depend on members of the karyopherin  [ **KPNA2** ]   [ **KPNB1** ]  family of Ran dependent nucleocytoplasmic transport factors for their nuclear export.  Ref: 12538759 J Cell Sci, 2003
- To further our understanding of how the nucleocytoplasmic transport machinery interfaces with its cargoes and how this affects cellular physiology, we investigated the molecular mechanisms of phenotypes associated with mutations in karyopherin  [ **KPNA2** ]   [ **KPNB1** ]  Kap121p.  Ref: 11909949 Mol Cell Biol, 2002
- We have identified a novel human karyopherin  [ **KPNA2** ]   [ **KPNB1** ]  Kap beta family member that is related to human Crm1 and the Saccharomyces cerevisiae protein, Msn5p Kap142p.  Ref: 11777942 J Cell Biol, 2002
- We have now developed a homogeneous solution based assay and show that NF kappa B interacts with karyopherin  [ **KPNA2** ]   [ **KPNB1** ]  alpha.  Ref: 12504098 Biochem Biophys Res Commun, 2003
